# Sex differences in cardiovascular morbidity associated with familial hypercholesterolaemia: A retrospective cohort study of the UK Simon Broome register linked to national hospital records

**DOI:** 10.1016/j.atherosclerosis.2020.10.895

**Published:** 2020-12

**Authors:** Barbara Iyen, Nadeem Qureshi, Stephen Weng, Paul Roderick, Joe Kai, Nigel Capps, Paul N. Durrington, Ian FW. McDowell, Handrean Soran, Andrew Neil, Steve E. Humphries

**Affiliations:** a Primary Care Stratified Medicine Group, Division of Primary Care, University of Nottingham, UK; b Faculty of Medicine, Primary Care and Population Sciences, University of Southampton, UK; c Department of Clinical Biochemistry, The Shrewsbury and Telford Hospital NHS Trust, Princess Royal Hospital, Telford, UK; d Cardiovascular Research Group, School of Clinical and Laboratory Sciences, University of Manchester, UK; e Department of Medical Biochemistry and Immunology, University Hospital of Wales, Cardiff, UK; f Centre for Diabetes, Endocrinology and Metabolism, Manchester University Hospitals NHS Foundation Trust, Manchester, UK; g Wolfson College, University of Oxford, UK; h Centre for Cardiovascular Genetics, Institute of Cardiovascular Science, University College London, University Street, London, UK

**Keywords:** Heterozygous familial hypercholesterolaemia, Cardiovascular disease, Gender

## Abstract

**Background and aims:**

The UK Simon Broome (SB) familial hypercholesterolaemia (FH) register previously reported 3-fold higher standardised mortality ratio for cardiovascular disease (CVD) in women compared to men from 2009 to 2015. Here we examined sex differences in CVD morbidity in FH by national linkage of the SB register with Hospital Episode Statistics (HES).

**Methods:**

Of 3553 FH individuals in the SB register (aged 20–79 years at registration), 2988 (52.5% women) had linked HES records. Standardised Morbidity Ratios (SMbR) compared to an age and sex-matched UK general practice population were calculated [95% confidence intervals] for first CVD hospitalisation in HES (a composite of coronary heart disease (CHD), myocardial infarction (MI), stable or unstable angina, stroke, TIA, peripheral vascular disease (PVD), heart failure, coronary revascularisation interventions).

**Results:**

At registration, men had significantly (*p* < 0.001) higher prevalence of previous CHD (24.8% *vs* 17.6%), previous MI (13.2% *vs* 6.3%), and were commenced on lipid-lowering treatment at a younger age than women (37.5 years *vs* 42.3 years). The SMbR for composite CVD was 6.83 (6.33–7.37) in men and 7.55 (6.99–8.15) in women. In individuals aged 30–50 years, SMbR in women was 50% higher than in men (15.04 [12.98–17.42] *vs* 10.03 [9.01–11.17]). In individuals >50 years, SMbR was 33% higher in women than men (6.11 [5.57–6.70] *vs* 4.59 [4.08–5.15]).

**Conclusions:**

Excess CVD morbidity due to FH remains markedly elevated in women at all ages, but especially those aged 30–50 years. This highlights the need for earlier diagnosis and optimisation of lipid-lowering risk factor management for all FH patients, with particular attention to young women with FH.

## Highlights

• Males with familial hypercholesterolaemia (FH) had more cardiovascular disease (CVD) risk factors and were commenced on lipid-lowering therapy at a younger age, than females with FH.
• Individuals with FH had excess rates of hospitalisations for CVD, compared to the general population of non-FH individuals.
• Excess CVD morbidity due to FH was higher in women than men aged 30 years and over, but especially in those aged 30–50 years.

## Graphical abstract

**Figure:**
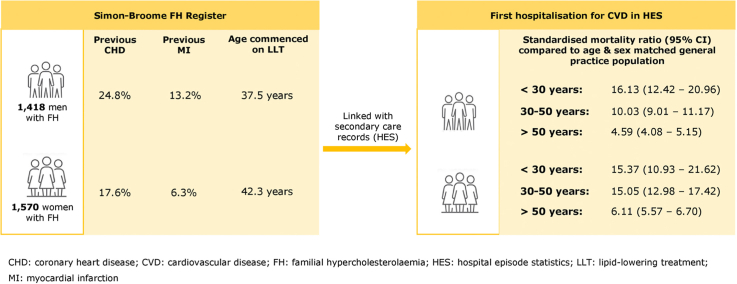

## 1 Introduction

Familial hypercholesterolaemia is an autosomal dominant disorder characterised by lifelong elevated plasma levels of low-density lipoprotein (LDL) cholesterol, which when untreated, leads to increased risk of coronary heart disease (CHD) and premature death [1]. More recently, individuals with FH phenotype in primary care have been shown to have a greatly increased risk of not only CHD, but also stroke and peripheral vascular disease (PVD) [2]. Heterozygous FH affects 1 in 250 to 1 in 300 of the general population but the majority of these individuals are undiagnosed [3,4]. If untreated, men with FH have a 50% risk of fatal or non-fatal CHD by age 50 years and women have a 30% risk by age 60 years [5].

In early studies of FH in the pre-statin era, CHD mortality was shown to be highest in the 20–39 year age group and reduced with increasing age [6]. Previous studies of the UK Simon Broome register examined changes in CHD mortality in FH patients both before and after the routine use of statins, and found that while there was a significant reduction in CHD mortality in men over time, excess mortality persisted in women [7], such that excess CHD mortality was 3-fold higher in women than men in the period from 2009 to 2015 [7]. These previous studies were limited to only coronary disease mortality outcomes in this patient population. As individuals with FH are now living longer due to effective lipid lowering therapies and coronary interventions [8], morbidity across a range of CVD outcomes now need to be examined. By linking records of Simon Broome register participants with their secondary care records from hospital episode statistics, we have now created the longest prospective cohort of FH patients known internationally.

This study evaluated the long-term CVD outcomes of individuals with heterozygous FH, and investigated any potential sex differences in cardiovascular disease morbidity associated with FH.

## 2 Materials and methods

### 2.1 Data source and baseline measures

The Simon Broome register includes individuals with FH recruited from 21 participating lipid clinics in the United Kingdom, to which they had been referred by either their general practitioners or hospital specialists. Recruitment of patients into the register began in 1980, and methods have been described previously [6,7,9]. Information recorded on registration into the Simon Broome register include individuals' baseline demographic and clinical characteristics such as age, smoking status, alcohol consumption, past medical history, medication history including use of lipid-lowering, antihypertensive and diabetic treatments, family history as well as clinical examination findings such as blood pressure, body mass index, tendon xanthomas, xanthelasma and arcus cornealis. A fasting blood specimen taken at the registration visit determined serum total cholesterol, triglycerides and high density lipoprotein [6,7,9]. Serum low density lipoprotein cholesterol (LDL-C) concentrations were calculated using the Friedewald equation [10]. Lipoprotein (a) (Lp(a)) concentration was measured in a single laboratory using a previously described method [11]. Patients were classified as having either Simon Broome Definite FH or Possible FH using previously published criteria [6,7,9]. Registered patients in the Simon Broome register were linked with the National Health Service Central Registry, which is part of the Office for National Statistics, for ascertainment of death records including underlying cause and date of death. For the current analysis, patients’ records have been linked to Hospital Episodes Statistics (HES) for ascertainment of secondary care inpatient morbidity data including admissions for cardiovascular disease. All patients were followed up from the date of their SB registration until their first hospitalisation for cardiovascular disease, date of death, emigration/loss to follow-up or last date of data collection, whichever occurred first. All patients gave informed consent for inclusion in the Simon Broome Register. The study received approval from the local ethics committee of each participating centre, and approvals for obtaining the linked hospital data was obtained by the NHS digital (DARS ref: NIC-115405) and Confidentiality Advisory Committee (CAG ref: 18/CAG/0007). The overall study obtained ethical approval from the NHS Health Research Authority (IRAS ref: 214219).

### 2.2 Cardiovascular disease outcome measures

Incident cardiovascular disease (CVD) was defined as the first hospital admission recorded in HES for coronary heart disease (CHD), myocardial infarction (MI), angina (stable or unstable), stroke, transient ischaemic attack (TIA), peripheral vascular disease (PVD), heart failure, or coronary revascularisation interventions such as percutaneous coronary interventions (PCI) or coronary artery bypass graft (CABG). Cardiovascular disease outcomes were identified from HES using the relevant ICD-10 and OPCS codes (shown in Supplementary Data).

### 2.3 Statistical analyses

Baseline characteristics of patients in the Simon Broome register were assessed, and these were reported as proportions, mean (standard deviation) and median (interquartile range) for categorical, continuous normally-distributed and continuous non-normally distributed variables, respectively. Appropriate statistical tests such as chi-squared, t-tests and Mann-Whitney U tests were used to assess differences in categorical and continuous variables, between males and females. Incidence rates of composite CVD outcomes were assessed for all SB patients as well as within pre-defined patient subgroups, and Cox-proportional hazard models estimated hazards ratios for CVD. We determined the observed number of incident CVD events per person-years of follow-up, stratified by sex and age-groups (<30 years, 30 to >50 years and in the over 50 year age-groups). Standardised morbidity ratios (SMbR) were calculated using indirect standardisation, with age and sex-specific CVD incidence rates of the UK primary care non-FH population, as reference rates [2]. We calculated the expected number of CVD events as the number of person-years of follow-up in the SB cohort multiplied by the incidence rate for the comparable age-group and sex in the reference population. Standardised morbidity ratios (SMbR) were computed as the observed number of CVD events divided by the expected number of events:

$$\text{SMbR}=\frac{\text{Σdi}}{\text{ΣEi}}=\frac{observed\;number\;of\;CVD\;events\;in\;the\;SB\;population}{\begin{array}{c} expected\;number\;of\;CVD\;events\;if\;the\;age-sex\;specific\;rates\;were\;the\;same\;as\;the\;\\ reference\;population \end{array}}$$

The 95% confidence intervals of the SMbR were derived using an error factor (EF), with the equation: 95% CI = SMbR / EF to SMbR × EF, where EF = exp (1.96 / √di).

SMbR was estimated for both composite CVD and constituent CVD outcomes. We conducted the primary analyses on all eligible individuals in the Simon Broome register, with or without a history of CVD. To evaluate the impact of having a previous history of CVD, we conducted sensitivity analyses by restricting the population to a subset of patients who had no history of previous CVD at the time of registration. Considering that secondary care records in HES only became available after 1 April 1997, a further sensitivity analysis was done with analyses restricted to only those individuals with registration dates in Simon Broome on or after 1 April 1997. All analyses were conducted using Stata SE version 15 statistical package.

## 3 Results

A total of 3553 subjects in the Simon Broome (SB) database were recruited from participating lipid clinics between 1 January 1980 and 20 December 2010. Of these, 2988 (84%) had linked HES admitted patient care records, and comprised the final study cohort. Individuals without linked HES records had comparable baseline demographic characteristics to those with linked data, but a higher proportion of them had records of previous history of CVD. A comparison of baseline characteristics between individuals with and without linked HES records is shown in Supplementary Table 1.

The characteristics of the study population, at the time of registration into the Simon Broome register, are shown in Table 1. Of the cohort, 1418 (47.5%) were male. Compared to men, women were 5 years older at registration and 4.8 years older at the time of commencing lipid lowering treatment (LLT). While women had a slightly lower BMI than men, their mean untreated total cholesterol concentration was significantly higher, but median triglyceride concentration was significantly lower. Consumption of alcohol was significantly higher in men than women but significantly fewer women reported ever smoking, while the prevalence of current smoking was similar in men and women. Fewer women reported a prior history of CVD than men, with significant difference in myocardial infarction (MI), CHD and previous revascularisation, and women having had their first MI 8 years later than men. While significantly more women than men had a history of hypertension, the prevalence of type 2 diabetes was similar in men and women, and although low overall, was similar to the prevalence of type 2 diabetes in the UK general population during the period of recruitment of the patient cohort [12]. Only 21.8% of the SB cohort had measures of Lp(a) at registration, but there was no statistically significant difference between these measures in males and females. Genetic test results were only available for 599 patients (20% of the study population) and an FH-causing variant was identified in 399 (67% of those tested), with no significant difference between males and females. Overall, women on the SB register had a better CVD risk factor profile, but a later age at FH diagnosis and commencement of LLT.

**Table 1:** Baseline characteristics of subjects with FH in the Simon Broome register (n = 2988).

		Male n (%) 1418 (47.46)	Female n (%) 1570 (52.54)	*p*-value^a^
Age at registration (years)	mean (SD)	41.1 (15.0)	46.1 (16.8)	<0.0001
BMI at registration (Kg/m^2^)	mean (SD)	25.17 (4.1)	24.78 (5.2)	0.0343
Follow-up (years)	median (IQR)	17.93 (11.17–23.98)	18.15 (11.59–23.73)	0.5993
FH diagnosis type		n = 1418	n = 1570	
Definite FH	n (%)	770 (54.3)	814 (51.9)	0.179
Possible FH		648 (45.7)	756 (48.1)	
Age started on LLT (years)	mean (SD)	37.5 (14.7)	42.3 (17.0)	<0.0001
Pre-treatment cholesterol (mmol/l)	mean (SD)	9.4 (2.8)	9.7 (2.0)	0.0136
Pre-treatment triglyceride (mmol/l)	median (IQR)	1.8 (1.2–2.7)	1.4 (1.0–2.2)	<0.0001
Pre-treatment lipoprotein Lp(a) (mg/dl)	median (IQR)	29 (10–63), n = 314	25 (11–70), n = 339	0.9927
Alcohol consumption (units/week)	median (IQR)	10 (1–20)	2 (0–9)	0.0001
Cigarette smoke exposure				
Ever smoked cigarette (yes)	n (%)	638 (45.0%, n = 1418)	605 (38.6%, n = 1568)	0.001
Current cigarette smoker (yes)		224 (16.0%, n = 1404)	293 (18.8%, n = 1556)	0.116
History of previous cardiovascular disease				
Angina	n (%)	250 (17.8%, n = 1403)	226 (14.5%, n = 1554)	0.091
Myocardial infarction		187 (13.2%, n = 1418)	99 (6.31%, n = 1570)	<0.0001
Coronary heart disease (yes)		352 (24.8%, n = 1418)	276 (17.6%, n = 1570)	<0.0001
Stroke (Yes)		10 (0.7%, n = 1404)	20 (1.3%, n = 1558)	0.173
Transient ischaemic attack		13 (1.3%, n = 1027)	18 (1.5%, n = 1168)	0.254
History of claudication		38 (2.7%, n = 1402)	49 (3.2, n = 1556)	0.790
Previous revascularisation (Angioplasty/CABG)		174 (17.0%, n = 1025)	96 (8.3%, n = 1161)	<0.0001
Age at first MI (years)	median (IQR)	43 (37–49)	51 (44–58.5)	0.0001
History of hypertension	n (%)	111 (10.9%, n = 1021)	196 (16.9%, n = 1162)	<0.0001
History of diabetes	n (%)	20 (1.4%, n = 1418)	19 (1.2%, n = 1570)	0.718
Use of other medications				
Beta-blockers	n (%)	117 (11.4%, n = 1028)	148 (12.7%, n = 1168)	0.644
Ace-inhibitors		39 (6.6%, n = 587)	54 (7.8%, n = 697)	0.610
Anti-platelet medication		257 (18.1%, n = 1418)	234 (14.9%, n = 1570)	0.010
Anticoagulant medication		9 (1.5%, n = 587)	10 (1.4%, n = 697)	0.830
Other antihypertensive medications		49 (4.8%, n = 1027)	74 (6.3%, n = 1168)	0.274
Type of FH mutation		n = 295	n = 304	
LDL-receptor	n (%)	178 (60.3%)	193 (63.5%)	0.416
Apo-B		10 (3.4%)	13 (4.3%)	
PCSK9		4 (1.4%)	1 (0.3%)	
None		103 (34.9%)	97 (31.9%)	

Tests of significance for categorical variables were derived using the Pearson's χ^2^ test.

a Independent *t*-test was used for comparison between continuous variables with normal distribution, and Mann-Whitney *U* test for variables with non-normal distribution.

### 3.1 Cardiovascular disease outcomes

Admitted patient care records from HES were available from April 1997 to March 2018. The median follow-up for patients in the SB register was 18.1 years (IQR 11.4–23.9), constituting 52,000 person-years of follow-up. Over this period, there were 1327 CVD-related hospital admissions. As shown in Table 2, the overall incidence rate for any CVD event in the SB patients was 25.47 (95% CI 24.14–26.88) per 1000 p-years follow-up. Incidence rates were lower in women, and compared to men, women had an adjusted hazard ratio (HR) of 0.65 (0.58–0.73). As expected, incidence rates and hazards ratio for CVD increased steeply with increasing age, with incidence rates ranging from 6.31 (5.12–7.77) in those less than 30 years at registration to 77.35 (59.67–100.28) in those over 70 years. The median age at first CVD-related hospital admission for CVD was 60.6 years (IQR 51.5–69.5) in men, and 70.0 years (IQR 60.0–77.6) in women. There were very few individuals with FH genetic test results, so statistically significant difference in hazard ratios for CVD could not be determined between the different FH-mutation carrier groups (data not shown). As expected, the CVD incidence rate was 4.5 fold higher in those with a previous history of CVD on registration, with an age and sex adjusted HR of 3.45 (3.06–3.89).

**Table 2:** Incidence rate and hazards ratios for composite CVD outcomes among SB patient population.

	Number of subjects	CVD events	Persons_years of follow-up	Incidence rate (95% CI) of CVD	Unadjusted hazards ratio for CVD	Adjusted HR for age and sex^b^
All subjects in SB register	2988	1327	52,090	25.47 (24.14–26.88)		
Sex						
Male	1418	669	24,630	27.17 (25.18–29.30)	1.00	1.00
Female	1570	658	27,470	23.96 (22.19–25.86)	0.89 (0.80–0.99)	0.65 (0.58–0.73)
Age at registration (years)						
<30	614	89	14,100	6.31 (5.12–7.77)	1.00	1.00
30 - <40	568	204	10,950	18.64 (16.25–21.38)	3.38 (2.63–4.34)	3.32 (2.59–4.26)
40 - <50	620	305	10,770	28.31 (25.30–31.67)	5.50 (4.34–6.98)	5.44 (4.29–6.90)
50 - <60	674	379	10,440	36.29 (32.81–40.13)	8.09 (6.40–10.23)	8.54 (6.75–10.80)
60 - <70	425	293	5090	57.51 (51.29–64.49)	16.21 (12.68–20.74)	17.88 (13.96–22.91)
>70	87	57	740	77.35 (59.67–100.28)	26.16 (18.58–36.84)	29.90 (21.18–42.20)
FH diagnosis type						
Definite FH	1584	726	28,150	25.79 (23.99–27.74)	1.00	1.00
Possible FH	1404	601	23,950	25.10 (23.17–27.18)	1.00 (0.90–1.12)	0.84 (0.75–0.93)
Past history of CVD						
No past history of CVD	2290	720	43,900	16.40 (15.25–17.64)	1.00	1.00
Past history of CVD	698	607	8190	74.10 (68.44–80.24)	5.90 (5.27–6.60)	3.45 (3.06–3.89)

Previous CVD includes previous CHD, MI, coronary revascularisation interventions (coronary angioplasty or CABG), stroke, TIA, intermittent claudication.

b HR for CVD in males and females was adjusted for age only, and HR for CVD in different age categories was adjusted for sex only.

**Fig. 1:**
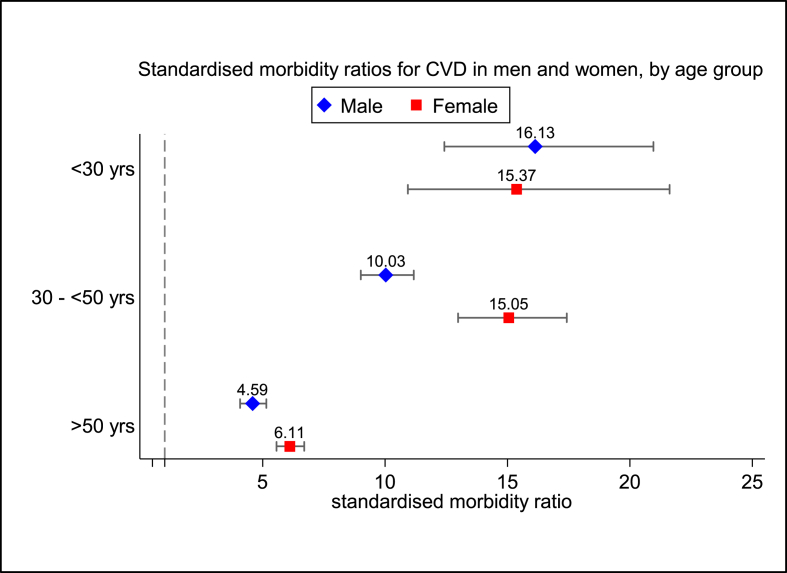
Standardised morbidity ratios for composite CVD in men and women with familial hypercholesterolaemia in the Simon Broome register.

Table 3 shows the observed number of CVD events across different age-groups in men and women in the SB register, as well as the number of CVD events that would be expected if these individuals had the same age- and sex-specific CVD incidence rates as the general practice population of individuals without FH. The overall standardised morbidity ratio (SMbR) among individuals with FH in the SB register was 7.17 (6.79–7.56). In both sexes, SMbR decreased with advancing age such that the highest excess CVD morbidity was in those younger than 30 years, and the lowest was in those over 50 years. Women with FH were observed to have larger excess CVD morbidity than men (7.55 (6.99–8.15) *vs* 6.83 (6.33–7.37) respectively). There were substantially significant sex differences in SMbR in patients aged 30–50 years and those older than 50 years (Fig. 1). These differences were most marked in the 30–50 year age group such that women had a 50% higher SMbR than men of the same age group (15.04 (12.98–17.42) *vs* 10.03 (9.01–11.17). In those older than 50 years, SMbR was 33% higher in women than men (6.11 (5.57–6.70) *vs* 4.59 (4.08–5.15) respectively). The median age at first hospitalisation for CVD in males and females with FH, by age-group at time of Simon Broome registration, is shown in Supplemental Table 2.

**Table 3:** Observed and expected number of CVD events in men and women with FH in the Simon Broome register.

	Person-years of follow-up	Observed CVD events	Incidence rates/1000 person years (95% CI)	Expected CVD events^a^	Standardised morbidity ratio (95% CI)
Males					
<30 years	6942	56	8.07 (6.21–10.48)	3.47	16.13 (12.42–20.96)
30 to <50 years	12,174	331	27.19 (24.41–30.28)	32.99	10.03 (9.01–11.17)
50 years	5510	282	51.18 (45.54–57.51)	61.49	4.59 (4.08–5.15)
Total (men)	24,627	669	27.17 (25.18–29.30)	97.96	6.83 (6.33–7.37)
Females					
<30 years	7157	33	4.61 (3.28–6.49)	2.15	15.37 (10.93–21.62)
30 to <50 years	9546	178	18.65 (16.10–21.60)	11.84	15.04 (12.98–17.42)
>50 years	10,765	447	41.52 (37.85–45.56)	73.20	6.11 (5.57–6.70)
Total (women)	27,468	658	23.96 (22.19–25.86)	87.18	7.55 (6.99–8.15)
Overall	52,094	1327	25.47 (24.14–26.88)	185.14	7.17 (6.79–7.56)

a Expected CVD events derived by applying age and sex-specific CVD incidence rates in the UK general practice population of non-FH subjects [2], to the number of person-years of follow-up.

**Fig. 2:**
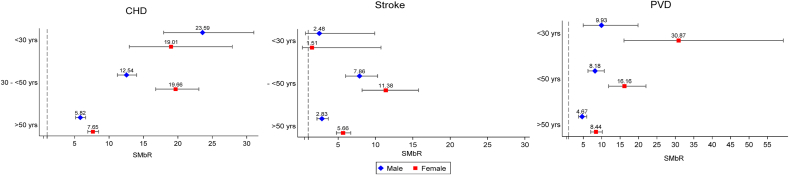
Standardised morbidity ratios for coronary heart disease, stroke, and peripheral vascular disease in males and females with familial hypercholesterolaemia.

On separate analyses of subtypes of the first CVD event, the SMbR for all subtypes were found to be higher in women than men (Fig. 2). The SMbR (95% CI) for CHD was substantially higher in women than men in the 30–50 year age group (19.66 (16.78–23.04) *vs* 12.54 (11.22–14.01)) and those over 50 years (7.65 (6.90–8.48) *vs* 5.82 (5.14–6.59)). Similarly, women had higher SMbR for PVD than men in the 30–50 year age group (16.16 (11.85–22.03) *vs* 8.18 (6.26–10.68)) and in the over 50 year age group (8.44 (7.02–10.14) *vs* 4.67 (3.68–5.93)). Higher SMbR for stroke was observed for women than men, but this was only in those aged over 50 years (5.66 (4.78–6.69) *vs* 2.83 (2.17–3.69)). In all CVD subtypes, the SMbR did not differ markedly between men and women with FH who were aged younger than 30 years (data are shown in Supplementary Table 3).

### 3.2 Sensitivity analyses

On restricting all analyses to only the subset of FH patients who had no history of previous CVD at the time of registration into the Simon Broome register, as expected, SMbR for men and women of all age groups were lower than estimates from the whole cohort. There were however similar findings of higher SMbR for CVD in women than men in the 30–50 year group (10.68 (8.88–12.86) *vs* 7.06 (6.12–8.14)) and in those aged 50 years and over (4.11 (3.61–4.68) *vs* 2.73 (2.26–3.31)) (Supplementary Table 4).

Further sensitivity analyses, which included only individuals who registered in the Simon Broome on or after the 1 April 1997, when secondary care records in HES were available, found that similar to results of analyses of the whole cohort, SMbR for CVD was higher in women than men. Unlike findings from the whole cohort, the higher SMbR in women compared to men was most marked in those younger than 30 years. Due to the small sample size and few observed number of CVD events in this subgroup of patients, there was insufficient power to detect statistically significant measures of effect, and so the 95% confidence intervals for these estimates were very wide (Supplementary Table 5).

## 4 Discussion

### 4.1 Main findings

This study of the 36 year prospective cohort of patients with FH has shown that patients living with FH had excess rates of hospitalisations for CVD across all age groups, compared to the general population of individuals without FH. As expected, the incidence rate of CVD was lowest in those who were under 30 years at time of registration, and this increased with increasing age. However, compared to the general non-FH population, excess CVD morbidity due to FH, was highest in the youngest age group and decreased with increasing age. Overall, SMbR was 16-fold higher in FH patients than the general population in those under 30 years, and was 5-fold higher in those over 50 years. At registration, men with FH had higher prevalence of CVD risk factors, and were diagnosed and commenced on lipid-lowering treatment earlier than women with FH. Although the absolute incidence rate of CVD associated with FH was higher in men than women across all age groups, excess CVD morbidity compared to the general population without FH was substantially higher in women than men aged 30 years and over, and this was most marked in those aged 30–50 years at time of registration in Simon Broome.

### 4.2 Strengths and limitations

The Simon Broome register is a well-established FH register, and until now it had been only linked to the Office for National Statistics for ascertainment of death records. This study used a new national linkage of patient records in the register with their secondary care records in hospital episode statistics, providing an 18-year follow-up, and making this the longest prospective study of FH in the world. To our knowledge, this study is the first to investigate long-term secondary care outcomes in a registry cohort of patients with FH. Over 84% of patients in the Simon Broome register had linked secondary care records from HES, which enabled comprehensive and robust ascertainment of different CVD outcomes, as well as interventions such as coronary interventions. We were also able to quantify the rates of coronary revascularisations in FH patients, which to date, has largely been understudied [13].

Study limitations include the lack of data on lipid-lowering treatments or more recent LDL-cholesterol concentrations beyond the time of SB registration, which could potentially explain the observed differences in CVD outcomes in men and women. It had however been shown in the 2010 survey of FH management in adults attending UK lipid clinics [14] that 86% of patients were on statin treatment, with 40% additionally being treated with Ezetimibe; which was associated with a median LDL-cholesterol reduction of 47% from baseline, at patients’ third lipid clinic visit [14]. As patients in our SB register cohort were recruited from UK lipid clinics, it is reasonable to assume that the proportion on lipid-lowering treatments will be consistent with findings from the audit, as well as more recent national guideline recommendations [15]. Over 50% of men and women in the SB register fulfilled the “definite FH” criteria, as such there may be a degree of selection bias towards those with more severe FH phenotype. We had no data on obstetric outcomes of women in this study, and so were unable to ascertain whether CVD outcomes differed between women with documented obstetric admissions who may have discontinued statin therapy during pregnancy and lactation, and those with fewer or no documented obstetric admissions. Also, electronically linked hospital records were only available from 1997 onwards, and therefore in participants who were registered prior to this date, there is likely under-ascertainment of CVD outcomes. Although our expectations are that any under-ascertainment of CVD outcomes are conservative in nature and not likely to affect males and females differently, we conducted a sensitivity analyses of those individuals whose date of registration in SB register was after the inception of HES on 1 April 1997. Although the finding of higher SMbR for CVD in women compared to men was broadly similar to findings from the main analyses, statistical significance could not be determined due to the small sample size of this patient subgroup.

A final limitation of the study is that, because DNA testing is not widely available in the UK, genetic testing was only done for 20% of the SB cohort, and only 13% of the total cohort had a DNA confirmed diagnosis. Therefore, there were too few mutation carriers to enable a statistically robust assessment of the relationship between mutation positive status (gene mutation) and CVD morbidity. However, all patients in the SB register were from specialist clinics and have clearly defined clinical phenotypes of FH.

### 4.3 Comparison with existing literature

Our study finding that excess CVD morbidity due to FH was highest in those younger than 30 years, and declined with advancing age, builds on previous research from the Simon Broome register, which reported the highest excess mortality from CHD before the age of 40 [6]. Consistent with our study findings, individuals in a Norwegian FH registry had the highest excess risk of acute MI and CHD in those aged 25–39 years [16].

The substantially higher SMbR for composite CVD in women than men, in the 30 to 50, and over 50 age groups, is a novel finding of considerable clinical significance. Although a study of the general population of adults with phenotypic FH in the United States had shown that age-based acceleration of CHD risk was greater for women than men [17], the study did not explore sex-differences in the increased risk of composite CVD outcomes associated with FH. It had previously been shown in the Simon Broome register that excess CHD deaths associated with FH were 3-fold higher in women than men for the period 2009 to 2015 [7]. While excess CHD deaths declined in men comparing the period before and after the routine use of statins, no corresponding decline was observed over time in women [7]. There are several possible explanations which may underlie our study finding of higher excess CVD morbidity in women compared to men. We found that the mean ages for commencing lipid-lowering treatment in men and women were 37.5 years and 42.3 years, respectively. While women typically develop CVD at a later age than men, the risk of CVD is often underestimated in women due to the misperception that females are ‘protected’ against CVD before the menopause [18]. While premenopausal women have a less pro-atherogenic plasma lipid profile than men, specifically greater high-density lipoprotein (HDL) and lower LDL-cholesterol than men of the same age [19], a study of children and adolescents with untreated FH suggests that the cholesterol burden with untreated FH is significantly higher in girls than boys [20]. These factors are likely to contribute to the greater excess CVD burden in women with FH compared to women without FH. Since statins are not recommended during pregnancy and breastfeeding, women with children are also likely to have experienced one or more interruptions of 2–3 years in their lipid lowering therapy and thus to have accumulated a greater “LDL-C burden” than men of the same age. Despite the greater CVD burden observed in women with FH compared to non-FH women, the incidence of CVD in individuals with FH remained lower in women than men across all age groups, suggesting that women may still retain some relative protection from CVD.

Statin prescribing rates have been shown to be lower in women than men in the general population [21], with numerous studies reporting that women in general are less likely to be prescribed evidence-based guidelines, and less aggressively treated in cardiology care for both primary [22] and secondary prevention [23]. Studies in the FH population have also demonstrated greater prescribing of more potent lipid-lowering therapy in men than women with FH, suggesting that FH treatment may be suboptimal in women [24,25]. This is consistent with findings from the survey of FH patients in UK lipid clinics, which showed that more men than women attained the FH treatment target of 50% or more reduction in baseline LDL-C [14]. In addition, gender was not found to be associated with adherence to statin therapy in a study of patients with FH [26].

### 4.4 Clinical implications and conclusion

This study finding of significantly higher risk of CVD in all age groups of patients in this registry-based cohort, compared to the general population, emphasizes the importance of early diagnosis and treatment of FH. Strategies to identify individuals with FH at a young age, before the development of significant coronary atherosclerosis, would be particularly helpful. Such strategies include cascade testing of relatives from identified index cases [27], and universal screening for high cholesterol in children, which would enable identification of parents with FH [28]. Our study provides confirmatory evidence of higher excess CVD morbidity in younger age groups of patients with FH and, importantly, provides novel insight into gender differences in the diagnosis and management of FH, as well as substantial gender disparities in the excess cardiovascular disease burden associated with FH. The finding that excess CVD morbidity is markedly higher in women than men in the 30–50 year age group, and also in those over 50 years, emphasizes the importance of early initiation of high intensity lipid-lowering treatment and highlights the need for optimisation of lipid-lowering and risk factor management for all FH patients with particular attention to women with FH.

## Author contributions

BI conducted the analysis, and BI and SEH wrote the first draft and subsequent revisions, led data management, and interpretation of findings. SW developed the study design, obtained ethical and study approvals to access the data, linked the SB-HES data, and cleaned the data into an analysable form. NQ and JK conceptualised the study design and methods, and provided primary care interpretation of findings. PR advised on refining study design, covariate selection. NQ and SEH provided overall supervision of the project. SEH, AN, PD, IFWM, NC and HS provided expert interpretation of study findings and formulated rationale for additional analyses. SW verified all statistical analyses. NQ, SW, JK, SEH, PR secured grant funding. All study authors have contributed to interpretation, revising, writing and finalising the final submission version of the manuscript.

## Declaration of competing interest

NQ has received honoraria and travel costs for lectures, meetings and survey from AMGEN. SW reports grants from 10.13039/100000002National Institute for Health Research Health Technology Assessment Programme during the conduct of the study, personal fees from AMGEN, personal fees from Quealth Ltd outside the submitted work and is a member of Clinical Practice Research Datalink Independent Scientific Advisory Committee (ISAC). SEH reports grants from 10.13039/501100000274British Heart Foundation during the conduct of the study. The remaining authors have no competing interests.

## References

[1] Austin M.A., Hutter C.M., Zimmern R.L., Humphries S.E. (2004). Genetic causes of monogenic heterozygous familial hypercholesterolemia: a HuGE prevalence review. Am. J. Epidemiol..

[2] Iyen B., Qureshi N., Kai J., Akyea R.K., Leonardi-Bee J., Roderick P. (2019). Risk of cardiovascular disease outcomes in primary care subjects with familial hypercholesterolaemia: a cohort study. Atherosclerosis.

[3] Nordestgaard B.G., Chapman M.J., Humphries S.E., Ginsberg H.N., Masana L., Descamps O.S. (2013). Familial hypercholesterolaemia is underdiagnosed and undertreated in the general population: guidance for clinicians to prevent coronary heart disease : consensus Statement of the European Atherosclerosis Society. Eur. Heart J..

[4] Beheshti S.O., Madsen C.M., Varbo A., Nordestgaard B.G. (2020). Worldwide prevalence of familial hypercholesterolemia: meta-analyses of 11 million subjects. J. Am. Coll. Cardiol..

[5] Marks D., Thorogood M., Neil H.A.W., Humphries S.E. (2003). A review on the diagnosis, natural history, and treatment of familial hypercholesterolaemia. Atherosclerosis.

[6] (1991). Risk of fatal coronary heart disease in familial hypercholesterolaemia. Scientific Steering Committee on behalf of the Simon Broome Register Group. BMJ.

[7] Humphries S.E., Cooper J.A., Seed M., Capps N., Durrington P.N., Jones B. (2018). Coronary heart disease mortality in treated familial hypercholesterolaemia: update of the UK Simon Broome FH register. Atherosclerosis.

[8] Murano S., Shinomiya M., Shirai K., Saito Y., Yoshida S. (1993). Characteristic features of long-living patients with familial hypercholesterolemia in Japan. J. Am. Geriatr. Soc..

[9] Neil H.A., Hawkins M.M., Durrington P.N., Betteridge D.J., Capps N.E., Humphries S.E. (2005). Non-coronary heart disease mortality and risk of fatal cancer in patients with treated heterozygous familial hypercholesterolaemia: a prospective registry study. Atherosclerosis.

[10] Friedewald W.T., Levy R.I., Fredrickson D.S. (1972). Estimation of the concentration of low-density lipoprotein cholesterol in plasma, without use of the preparative ultracentrifuge. Clin. Chem..

[11] Neil H.A., Seagroatt V., Betteridge D.J., Cooper M.P., Durrington P.N., Miller J.P. (2004). Established and emerging coronary risk factors in patients with heterozygous familial hypercholesterolaemia. Heart.

[12] Sharma M., Nazareth I., Petersen I. (2016). Trends in incidence, prevalence and prescribing in type 2 diabetes mellitus between 2000 and 2013 in primary care: a retrospective cohort study. BMJ Open.

[13] Ungar L., Sanders D., Becerra B., Barseghian A. (2018). Percutaneous coronary intervention in familial hypercholesterolemia is understudied. Frontiers in Cardiovascular Medicine.

[14] Seed M., Roughton M., Pedersen K., Nair D., Wang T., Neil A. (2012). Current statin treatment, DNA testing and cascade testing of UK patients with familial hypercholesterolaemia. Primary Care Cardiovascular Journal.

[15] National Institute for Health and Care Excellence (2008). Familial hypercholesterolaemia: identification and management national Institute for Health and care excellence. NICE clinical guideline (CG71). https://www.nice.org.uk/guidance/cg71.

[16] Mundal L.J., Igland J., Veierod M.B., Holven K.B., Ose L., Selmer R.M. (2018). Impact of Age on Excess Risk of Coronary Heart Disease in Patients with Familial Hypercholesterolaemia.

[17] Perak A.M., Ning H., de Ferranti S.D., Gooding H.C., Wilkins J.T., Lloyd-Jones D.M. (2016). Long-term risk of atherosclerotic cardiovascular disease in US adults with the Familial Hypercholesterolemia phenotype. Circulation.

[18] Maas A.H.E.M., Appelman Y.E.A. (2010). Gender differences in coronary heart disease. Neth. Heart J..

[19] Wang X., Magkos F., Mittendorfer B. (2011). Sex differences in lipid and lipoprotein metabolism: it's not just about sex hormones. J. Clin. Endocrinol. Metabol..

[20] Holven K.B., Narverud I., van Lennep J.R., Versmissen J., Øyri L.K.L., Galema-Boers A. (2018). Sex differences in cholesterol levels from birth to 19 years of age may lead to increased cholesterol burden in females with FH. Journal of Clinical Lipidology.

[21] O'Keeffe A.G., Nazareth I., Petersen I. (2016). Time trends in the prescription of statins for the primary prevention of cardiovascular disease in the United Kingdom: a cohort study using the Health Improvement Network primary care data. Clin. Epidemiol..

[22] Lee S.K., Khambhati J., Varghese T., Stahl E.P., Kumar S., Sandesara P.B. (2017). Comprehensive primary prevention of cardiovascular disease in women. Clin. Cardiol..

[23] Redfors B. (2017). Women Are Less Likely to Get Secondary Prevention Medications and Cardiac Rehabilitation.

[24] Zamora A., Masana L., Comas-Cufi M., Vila A., Plana N., Garcia-Gil M. (2017). Familial hypercholesterolemia in a European Mediterranean population-Prevalence and clinical data from 2.5 million primary care patients. J Clin Lipidol.

[25] Amrock S.M., Duell P.B., Knickelbine T., Martin S.S., O'Brien E.C., Watson K.E. (2017). Health disparities among adult patients with a phenotypic diagnosis of familial hypercholesterolemia in the CASCADE-FH™ patient registry. Atherosclerosis.

[26] Korneva V., Kuznetsova T., Julius U. (2019). Efficiency and problems of statin therapy in patients with heterozygous familial hypercholesterolemia. Atherosclerosis Suppl..

[27] Kerr M., Pears R., Miedzybrodzka Z., Haralambos K., Cather M., Watson M. (2017). Cost effectiveness of cascade testing for familial hypercholesterolaemia, based on data from familial hypercholesterolaemia services in the UK. Eur. Heart J..

[28] Wald D.S., Bestwick J.P., Morris J.K., Whyte K., Jenkins L., Wald N.J. (2016). Child–parent familial hypercholesterolemia screening in primary care. N. Engl. J. Med..

