# Supplementary material for: Sex differences in cardiovascular morbidity associated with familial hypercholesterolaemia: A retrospective cohort study of the UK Simon Broome register linked to national hospital records
Source: Atherosclerosis. 2020 Dec;315:131–7. doi: 10.1016/j.atherosclerosis.2020.10.895 (PMC7754706; doi:10.1016/j.atherosclerosis.2020.10.895)
Supplement: Multimedia component 1 [file mmc1.docx]

**Supplementary tables**

Supplementary table 1. Baseline characteristics of individuals in SB with linked HES data and those without linked HES data

|  | unit | SB patients with linked HES data n (%)  2,988 (100) | Unlinked SB patients n (%)  565 (100) |
| --- | --- | --- | --- |
| Male | n (%) | 1,418 (47.46) | 297 (52.57) |
| Age (years) at registration | (mean(SD)) | 43.7 (16.2) | 44.2 (16.1) |
| BMI at registration (kg/m^2^) | (mean(SD)) | 24.97 (4.7) | 25.01 (4.1) |
| Follow-up (years) | (median (IQR)) | 18.07 (11.39-23.86) | 15.72 (7.90-23.54) |
| FH diagnosis type  Definite FH  Possible FH | n (%) | 1,584 (53.0)  1,404 (47.0) | 319 (56.5)  246 (43.5) |
| Age started on LLT (mean(SD)) | n (%) | 40.0 (16.1) | 40.1 (16.0) |
| Pre-treatment cholesterol (mmol/L) | (mean(SD)) | 9.6 (2.4) | 9.8 (2.2) |
| Pre-treatment triglyceride (mmol/L) | (median (IQR)) | 1.6 (1.1-2.5) | 1.6 (1.1-2.4) |
| Alcohol consumption (units/week) | (median (IQR)) | 4 (0-12) | 4 (0-10) |
| Cigarette smoke exposure  Ever smoked cigarette (yes)  Current cigarette smoker (yes) | n (%) | 1,243 (41.63)  517 (17.47) | 269 (47.70)  110 (19.93) |
| History of previous cardiovascular disease  Angina   - Definite - Possible   Myocardial infarction   - Definite - Possible   Coronary heart disease (yes)  Stroke (Yes)  Transient ischaemic attack  History of claudication  Previous revascularisation (Angioplasty/CABG) | n (%) | 476 (16.10)  85 (2.87)  286 (9.57)  50 (1.67)  628 (21.02)  30 (1.01)  31 (1.41)  87 (2.94)  270 (12.35) | 138 (25.00)  25 (4.53)  82 (14.51)  18 (3.19)  180 (31.86)  15 (2.70)  4 (1.20)  35 (6.38)  46 (8.14) |
| Age (years) of first MI | (median (IQR)) | 45 (38-53) | 46.5 (35-57.5) |
| History of hypertension | n (%) | 307 (14.06) | 47 (14.11) |
| History of Diabetes | n (%) | 39 (1.31) | 6 (1.06) |

Supplementary Table 2. Median age at first hospitalisation for CVD, by age group at time of registration in Simon Broome

| Age-category (years) at time of registration | Median age (IQR) at first CVD hospitalisation in HES | |
| --- | --- | --- |
|  | Males | Females |
| <30 | 44.0 (39.6-47.9) | 42.4 (38.3-49.1) |
| 30 to <50 | 54.8 (49.1-61.5) | 58.0 (50.4-66.2) |
| >50 | 70.1 (64.3-76.3) | 74.3 (68.5-80.4) |

Supplementary table 3. Observed and expected number of events of different CVD outcomes in males and females

|  | **Males** | | | | **Females** | | | |
| --- | --- | --- | --- | --- | --- | --- | --- | --- |
| Age category | P-yrs of follow up | Observed events | Expected events | Standardised morbidity ratio (95% CI) | P-yrs of follow up | Observed events | Expected events | Standardised morbidity ratio (95% CI) |
| Coronary heart disease | | | | |  |  |  |  |
| <30 years  30 to <50 years  >50 years | 6,970  12,380  5,620 | 51  312  250 | 2.16  24.89  42.96 | 23.59 (17.93-31.04)  12.54 (11.22-14.01)  5.82 (5.14-6.59) | 7,200  9,730  10,900 | 26  153  362 | 1.37  7.78  47.32 | 19.01 (12.94-27.92)  19.66 (16.78-23.04)  7.65 (6.90-8.48) |
| Total | 24,970 | 613 | 70.01 | 8.76 (8.09-9.48) | 27,827 | 541 | 56.47 | 9.58 (8.81-10.42) |
| Stroke | | | | |  |  |  |  |
| <30 years  30 to <50 years  >50 years | 7,341  14,994  7,089 | 2  53  55 | 0.81  6.75  19.42 | 2.48 (0.62-9.90)  7.86 (6.00-10.28)  2.83 (2.17-3.69) | 7,354  10,910  12,461 | 1  36  136 | 0.66  3.16  24.05 | 1.51 (0.21-10.73)  11.38 (8.21-15.77)  5.66 (4.78-6.69) |
| Total | 29,424 | 110 | 26.98 | 4.08 (3.38-4.92) | 30,723 | 173 | 27.88 | 6.21 (5.35-7.20) |
| Peripheral vascular disease | | | | |  |  |  |  |
| <30 years  30 to <50 years  >50 years | 7,321  15,006  7,031 | 8  54  68 | 0.81  6.60  14.55 | 9.93 (4.97-19.86)  8.18 (6.26-10.68)  4.67 (3.68-5.93) | 7,290  10,762  12,178 | 9  40  113 | 0.29  2.48  13.40 | 30.87 (16.06-59.32)  16.16 (11.85-22.03)  8.44 (7.02-10.14) |
| Total | 29,358 | 130 | 21.96 | 5.92 (4.98-7.03) | 30,230 | 162 | 16.16 | 10.02 (8.59-11.69) |

Supplementary Table 4. Sensitivity analysis showing observed and expected number of CVD events in men and women with FH and no previous CVD in the Simon Broome register

|  | Person-years of follow-up | Observed CVD events | Incidence rates / 1000 person years (95% CI) | Expected CVD events § | Standardised morbidity ratio (95% CI) |
| --- | --- | --- | --- | --- | --- |
| **Men** |  |  |  |  |  |
| <30 years  30 to <50 years  50 years | 6,910  9,880  3,410 | 55  189  104 | 7.96 (6.11-10.30)  19.13 (16.59-22.06)  30.51 (25.18-36.98) | 3.45  26.77  38.04 | 15.92 (12.23-20.74)  7.06 (6.12-8.14)  2.73 (2.26-3.31) |
| Total | 20,200 | 348 | 17.23 (15.51-19.14) | 68.27 | 5.10 (4.59-5.66) |
| **Women** |  |  |  |  |  |
| <30 years  30 to <50 years  >50 years | 7,070  8,450  8,190 | 31  112  229 | 4.39 (3.09-6.24)  13.25 (11.01-15.94)  27.97 (24.57-31.84) | 2.12  10.48  55.67 | 14.62 (10.28-20.79)  10.68 (8.88-12.86)  4.11 (3.61-4.68) |
| Total | 23,710 | 372 | 15.69 (14.18-17.37) | 68.27 | 5.45 (4.92-6.03) |

§ Expected CVD events derived by applying age and sex-specific CVD incidence rates in the UK general practice population of non-FH subjects (2), to the number of person-years of follow-up

Supplementary Table 5. Sensitivity analyses showing observed and expected number of CVD events in men and women who registered in the Simon Broome register on or after the 1^st^ of April 1997

|  | Person-years of follow-up | Observed CVD events | Incidence rates / 1000 person years (95% CI) | Expected CVD events § | Standardised morbidity ratio (95% CI) |
| --- | --- | --- | --- | --- | --- |
| **Males** |  |  |  |  |  |
| <30 years  30 to <50 years  50 years | 803  1,765  1,109 | 2  54  63 | 2.49 (0.62-9.96)  30.58 (23.42-39.93)  56.82 (44.39-72.74) | 0.40  4.79  12.37 | 4.98 (1.25-19.91)  11.29 (8.64-14.73)  5.09 (3.98-6.52) |
| Total (men) | 3,678 | 119 | 32.36 (27.04-38.72) | 17.56 |  |
| **Females** |  |  |  |  |  |
| <30 years  30 to <50 years  >50 years | 837  1,626  2,283 | 3  24  90 | 3.58 (1.16-11.11)  14.76 (9.90-22.03)  39.42 (32.06-48.46) | 0.25  2.02  15.53 | 11.94 (3.85-37.03)  11.91 (7.98-17.76)  5.80 (4.72-7.13) |
| Total (women) | 4,746 | 117 | 24.65 (20.57-29.55) | 17.79 | 6.58 (5.49-7.88) |
|  |  |  |  |  |  |

§ Expected CVD events derived by applying age and sex-specific CVD incidence rates in the UK general practice population of non-FH subjects (2), to the number of person-years of follow-up
